# Evidence of the Complexity of Gene Expression Analysis in Fish Wild Populations

**DOI:** 10.1155/2017/1258396

**Published:** 2017-10-23

**Authors:** Mbaye Tine

**Affiliations:** UFR des Sciences Agronomiques, de l'Aquaculture et des Technologies Alimentaires (UFR S2ATA), Universite Gaston Berger (UGB), Route de Ngallele BP 234, Saint-Louis, Senegal

## Abstract

The present work examines the induction of the *band 3 anion transport protein*, *mitogen-activated protein kinase*, and *lactate dehydrogenase*, respectively related to osmolyte transport, cell volume regulation, and energy production in the gills of two tilapia strains exposed to either freshwater or hypersaline water. Overall, genes showed similar expression patterns between strains. However, a wild population survey across a range of natural habitats and salinities did not reveal the expected patterns. Although significant, the correlations between gene expression and salinity were slightly ambiguous and did not show any link with phenotypic differences in life history traits previously reported between the same populations. The differential expression was also not associated with the population genetic structure inferred from neutral markers. The results suggest that the differential expression observed is not the result of evolutionary forces such as genetic drift or adaptation by natural selection. Instead, it can be speculated that genes responded to various abiotic and biotic stressors, including factors intrinsic to animals. This study provides clear evidence of the complexity of gene expression analysis in wild populations and shows that more attention needs to be paid when selecting candidates as potential biomarkers for monitoring adaptive responses to a specific environmental perturbation.

## 1. Introduction

Natural variations in environmental factors such as temperature, salinity, pH and dissolved oxygen concentration, and anthropogenic alterations of natural habitats are among the environmental stressors that have profound and diverse impacts on aquatic ecosystems [1, 2]. Climate change can lead to shifts in physicochemical factors that include salinity, temperature, dissolved oxygen levels, and water quality [3]. While salinity has been identified as a key environmental factor that may have physiological effects on aquatic species, temperature, dissolved oxygen, and water quality are also among the major environmental constraints that may have broad impact on fish biological functions such as growth and reproduction [4–6]. Changes in these physicochemical factors can be stressful for many organisms including fishes and can potentially induce adaptive stress responses [7, 8]. Organisms adapt to environmental changes prevailing in their natural environment through fixation of changes in protein-coding DNA sequences that alter protein function and enhance individual fitness and/or via epigenetic changes that affect gene expression [9–12].

Abrupt changes in ambient conditions require fast and specific responses of preexisting molecular pathways associated with acclimation responses. Compared to other vertebrates, fishes are characterized by very plastic phenotypic responses to changing environmental conditions, which are reflected as drastic and rapid changes in gene expression in response to the external stimuli. Therefore, one might expect changes in gene expression patterns to be in the same direction as the environmental factor that has induced them. Short-term physiological acclimation is facilitated by changes at the cellular level, which include the activation of pathways and the induction of candidate genes involved in the adaptation to the environmental stress. The modulation of gene expression is, therefore, among the first stress responses available to fishes that are confronted with changes in ambient conditions. Consequently, the expression and regulation of genes related to individual fitness have been widely used to investigate the adaptation of wild populations to local environmental conditions [13].

Experimental research generally uses common garden experiments to evaluate the role of gene expression in an organisms' response to changes in environmental conditions. Such experiments are helpful in order to distinguish environmental influences from genetic influences on phenotypic variations among populations. They have been successfully used in a range of taxa, including fishes (38–40). When conducted with offspring from a single population with the same genetic background, common garden experiments can also be used to reveal which among a suite of environmental factors is responsible for interindividual variation in a given phenotypic trait of a population [14]. This is achieved by varying one environmental parameter while keeping the others constant.

The strict control of environmental parameters in common garden experiments is one of the major differences compared with studies in species' native environments, where biotic and abiotic factors cannot be controlled. Comparative and evolutionary studies of wild populations are often conducted across environmental clines, such as temperature and salinity gradients [15–18]. This implies that there is at least one environmental factor that is the most constraining for the species in the study area and that predominates over other factors. However, despite this predominance, there are many other biotic/abiotic parameters whose variations, however slight, may directly or indirectly affect the expression of certain genes. The effects of such variables may interfere with the predominant factor depending on whether the genes analysed are involved in one or several biological pathways and also on the degree of the environmental stressor to which they respond.

Studies on osmoregulation have yielded a large amount of information on the cellular and molecular modifications associated with spatiotemporal changes in salinity [19–22]. Most of these studies have focused on water- and ion-transporting proteins [23–26], and the osmoregulatory roles of these ion pumps are now well understood at both cellular and molecular levels. Acclimation to spatiotemporal changes in salinity depends on the ability of the gill epithelium to adjust NaCl secretion or absorption, depending on environmental salinity [23, 26]. However, the regulation of ion concentrations is not the only challenge with which fish are confronted. They are also confronted with changes in cellular volume induced by salinity changes. Unfortunately, the mechanisms of cellular volume regulation remain poorly understood.

The black-chinned tilapia, *Sarotherodon melanotheron*, is a euryhaline teleost widely distributed in West African aquatic ecosystems, where it is regularly exposed to a wide range of salinity values. The species is found in marine coastal waters and freshwater habitats, where salinity is constant throughout the year [27]. It is also represented in estuaries, such as the Saloum estuary in Senegal, which exhibits extreme variations in salinity, ranging from fresh to extremely hypersaline (up to 130 psu), and where seasonal variations in salinity can be considerable [28, 29]. The existence of a salinity cline across the distributional range of the species offers an excellent opportunity for investigating gene expression responses to salinity changes.

The aim of the present study was to assess the relationship between salinity and gene expression patterns in three proteins, namely, *band 3 anion transport protein* (SLC4A1), *mitogen-activated protein kinase* (MAPK), and *lactate dehydrogenase* (LDH), in relation to osmolyte transport, cell volume regulation, and energy production, respectively, in individuals of the black-chinned tilapia acclimatised to different salinities. Based on their function, these genes may play important roles in *S. melanotheron* acclimatisation to salinity variations. The SLC4A1 is a transport protein that promotes the reversible exchange of bicarbonate ion for chloride ion [30]. This anion exchanger is the major contributor of the compensation mechanism of cell swelling because it facilitates the efflux of osmolytes, including KCl and amino acids [31, 32], a mechanism that is crucial in pH and cell volume regulation. MAPKs are protein kinases that respond to several extracellular signals and participate in the regulation of various cellular activities such as gene expression, mitosis, differentiation, and cell survival/apoptosis [33, 34]. MAPKs are involved in amplification and integration of extracellular signal and are, therefore, very important in the adaptation of fish to salinity. The LDH is a key enzyme of glycolysis involved in anaerobic energy production and reversible conversion of pyruvate and NADH into lactate and NAD^+^, respectively [35]. This enzyme is found abundantly in some organs including the heart, kidney, liver, muscle, and, to a lesser extent, gills, where it plays an important role in glucose homeostasis.

The three genes analysed in this study were initially identified using suppressive subtractive hybridisation (SSH) on *S. melanotheron* acclimatised to different salinities (0 and 70 psu) in controlled laboratory conditions [36]. They were selected for the current study because they are not housekeeping or reference genes, that is, genes expressed in a wide variety of tissues or cells with no or minimum variation in gene expression patterns between individuals. They were chosen based on the patterns of their expression response to ambient salinity in experimental conditions that is relevant to the ability of individuals to respond to environmental changes. Accordingly, their transcriptional responses are likely to reflect salinity differences in natural environments where this parameter is the predominant stressor.

We previously published previous works conducted on the same populations using genes encoding ion pumps and channels (Na/K-ATPase, voltage-dependent anion channel) [37, 38], genes encoding general stress proteins such as HSP70 [37] (from the same SSH libraries), and pituitary hormones (growth hormone, prolactin) [29]. There were clear relationships between the levels of mRNA expression of these genes and the environmental salinity, which can be explained by the fact that these genes play major roles in the responses to osmotic stress. For this present study, we sought to test other genes (those from the same SSH libraries rather than those analysed in the first published reports) whose involvement in osmoregulatory processes has not been yet or well described. We selected genes that may be involved in osmoregulation because their involvement in physiological processes related to this function such as osmolyte transport, energy production, and cell volume and signal regulation is commonly accepted. It is likely that these physiological processes are activated during adaptive responses to biotic or abiotic stressor other than ambient salinity. Although water salinity is the most constraining abiotic factor for *S. melanotheron* in our study areas, gene expression may be influenced by biotic stressors including factors intrinsic to animals and whose effects are difficult to evaluate in wild populations.

Relative levels of the expression of these genes in the fishes' gills were first compared under experimental conditions in individuals of *S. melanotheron* whose broodstock originated from two different strains that had been collected in hypersaline water and seawater, respectively. I then investigated the hypothesis that relative gene expression in wild populations acclimatised to salinities ranging from 0 to 100 psu is correlated with salinity. The new set of genes was analysed on samples collected at the same time rather than those used in our previously published studies. The main advantage of this study compared to that of previous works is the comparison of the transcriptional responses between conditions and strains, followed by a survey of wild populations across a range of natural habitats and salinities, which constitutes an excellent approach for selecting candidates as potential biomarkers for monitoring adaptive responses to a specific environmental perturbation. The combination of experimental and wild populations also offers the advantage of allowing a better understanding of the role of gene expression in fish adaptation to extreme salinities and could also highlight the complexity of gene expression analysis in natural environments.

## 2. Methods

### 2.1. Experiments Conducted under Controlled Conditions

**Figure 1:**
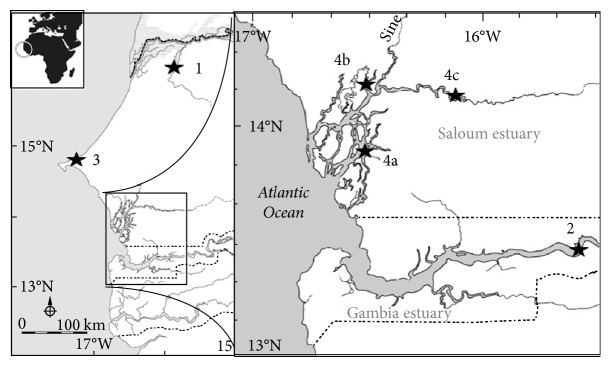
Map showing sampling locations. 1: Guiers Lake; 2: Balingho; 3: Hann Bay; 4a: Missirah; 4b: Foundiougne; 4c: Kaolack.

The original broodstock of the black-chinned tilapia (*S. melanotheron*) juveniles used in this study was collected by Dr. Abdou Mbow (IFAN) during the rainy season at Kaolack (site 4c in Figure 1) in the upper reaches of the Saloum estuary in Senegal, at a salinity of 48 psu. They were transferred to the CIRAD facilities (Montpellier) nearly 15 years ago. Fishes were transported under a joint collaborative project between IFAN and CIRAD research institutes, and no permit for transporting fish to France was required at that time. Since then, fish from the same initial broodstocks have been used in many scientific publications [36–39]. They were divided into two groups of 60 individuals each and maintained in 30 L freshwater aquaria for three days before being transferred to seawater. Each aquarium was equipped with a thermostat to maintain a 25°C constant water temperature (Table 1). The aquaria were equipped with a mechanical/biological filtration system and airstones to ensure optimal oxygen saturation. Fish were fed with commercial processed fish feed containing 40% crude proteins which was distributed six days a week. After an acclimation period of one week in seawater, the salinity at which half of the fish were held was increased to hypersaline conditions (70 psu) while in the other half, it was decreased to freshwater conditions. Salinity changes were carried out by slowly emptying the seawater and replacing it with either freshwater or hypersaline water. The commercial marine salt (Instant Ocean® salts) used to make seawater and hypersaline water contains all the components of natural seawater and was initially dissolved in a bucket with the same water as that used in the freshwater aquaria before being added to a particular aquarium. After 45 days, fish in freshwater and hypersaline water were anesthetized with 2-phenoxyethanol (1.5 mL/L of water), weighed, and killed by decapitation. Gill tissues were collected, immediately frozen in liquid nitrogen, and stored at −80°C until processing. The same procedure was followed for another black-chinned tilapia strain that was collected in seawater (Joal, Senegal). This second experiment was essentially conducted to validate the results from the first experiment. The genes that are differentially expressed in both strains as a result of salinity variations are most likely to be real candidates for salinity adaptation. The experimental conditions were identical to those described for the first experiment, except that the time of the year when the experiment was conducted, the size and type of the tanks, the type of food, and the developmental stage differed from those of the first experiment. In the second experiment, gill tissues were collected after an exposure period of only 10 days to freshwater and hypersaline water. This experiment was specially designed to identify genes involved in the long-term survival of fish exposed to specific salinity conditions. Such routinely expressed genes can be expected to be the target of adaptive selection in wild populations that are subjected to long-term natural acclimatisation. For this reason, samples were collected after an exposure period of 45 or 10 days to different conditions because at these times, fish can be considered already acclimatised to extreme salinities [40, 41].

### 2.2. Sampling Design of Natural Populations

Six natural populations (Figure 1) of *Sarotherodon melanotheron* were sampled in 2006 at the end of the dry season (May), when the salinity in the Saloum estuary was at its maximum level and has been stable for few months. Two of the sampling sites [Guiers Lake (site 1) and Hann Bay (3)] have relatively stable salinities throughout the year, while the other sites (three locations of the Saloum estuary, namely, Missirah (4a), Foundiougne (4b), and Kaolack (4c), and one location in the Gambia estuary, Balingho (2)) exhibit large spatial and seasonal variations in salinity. Fish sampling was carried out by local fishermen using cast nets. Only five fish were sampled from each cast net thrown in order to limit fish stress and prevent variability resulting from handling. Given that gene expression could conceivably be influenced by differences in the developmental or sexual stage, only size classes between 120 and 160 mm fork length with sexual stage 1 or 2 [42] corresponding to immature individuals were analysed (Table 2). All individuals were collected at the same time during the same sampling campaign and samples treated identically to avoid sampling artefacts. Gills were immediately extracted from these captured individuals and stored in RNAlater (Ambion) at 4°C for 24 h and then at −20°C until processing.

### 2.3. Ethical Statement

No ethical approval was required for this study. The samples used in this study were collected in accordance with good animal practice as outlined by the French Research Institute for Exploitation of the Sea (IFREMER) in a training course on how to handle fish and promote their welfare under experimental conditions. The IFREMER do not approve or give a permit for studies of wild populations of fish but only provide a code of conduct to follow to minimize the suffering in experiments involving fish. Study approval by another academic ethics committee (permit number or approval ID) was not necessary as all procedures carried out with the black-chinned tilapia in this study conformed to the IFREMER recommendations. No specific permissions were required for the collection of samples in the Saloum estuary (Kaolack, Foundiougne, and Missirah locations), Guiers Lake, and Hann Bay. These ecosystems are undergoing important environmental and anthropogenic perturbations. The Senegalese authorities encourage scientists to conduct scientific projects in these areas to better understand the consequences of these environmental and anthropogenic constraints on live history traits of organisms inhabiting these areas including fishes. To encourage them to investigate these areas, the government gave free access to scientists to perform their research without any prior administrative permission. The permission for sampling in Balingho location in the Gambia estuary was verbally given by the Gambia Fisheries Department. No official written document from the Gambian Fisheries Department was required. Likewise, approval for working on the tilapia *S. melanotheron* in the study area was not required from any animal ethics committee because this species is not an endangered species. Instead, it is among the most adapted to environmental and anthropogenic changes in the Senegal and Gambia estuaries, and scientists have convinced the authorities to use it as a model for biological and ecological studies.

### 2.4. Environmental Data Collection

For each sampling location, salinity (psu) and temperature (°C) were measured in situ at the time and place where fish were sampled with a refractometer (ATAGO) and a thermometer, respectively (Table 2). In the Saloum and Gambia estuaries, additional salinity, temperature, and dissolved oxygen data were obtained from experimental fish sampled by the IRD research group UR 070-RAP (Dakar, Senegal). During these sampling expeditions, surface salinity was measured with an optical refractometer and bottom salinity was measured with a YSI multiparameter probe that allows simultaneous measurements of salinity, water temperature, and dissolved oxygen. The same probe was used for the measurements of surface and bottom percentage oxygen saturation. Previous studies have demonstrated that there is no significant difference between the bottom and surface water temperatures in these sampling locations [4, 6]. Therefore, only the temperature near the water surface was measured with a thermometer. Additional data on salinity, water temperature, and dissolved oxygen were taken from the weather station of the IRD research group UR098-FLAG (Dakar, Senegal) for Guiers Lake and Hann Bay locations (Supplementary Table 1 in Supplementary Material available online at https://doi.org/10.1155/2017/1258396).

### 2.5. Selection of Candidate Markers

Based on their function, these genes may also play important roles in *S. melanotheron* acclimatisation to salinity variations. The SLC4A1 is a transport protein that promotes the reversible exchange of bicarbonate ion for chloride ion [30]. This anion exchanger is the major contributor of the compensation mechanism of cell swelling because it facilitates the efflux of osmolytes, including KCl and amino acids [31, 32], a mechanism that is crucial in pH and cell volume regulation. MAPKs are protein kinases that respond to several extracellular signals and participate in the regulation of various cellular activities such as gene expression, mitosis, differentiation, and cell survival/apoptosis [33, 34]. MAPKs are involved in amplification and integration of extracellular signal and are, therefore, very important in the adaptation of fish to salinity. The LDH is a key enzyme of glycolysis involved in anaerobic energy production and reversible conversion of pyruvate and NADH into lactate and NAD^+^, respectively [35]. This enzyme is found abundantly in some organs including the heart, kidney, liver, muscle, and, to a lesser extent, gills, where it plays an important role in glucose homeostasis.

There are no genes that are universally controlled by salinity. Therefore, positive controls (indicator of osmoregulation status) such as genes involved in ion uptake would be used to show a well-known salinity-regulated gene pattern. Such an indicator could be added to evaluate the stress status of fish induced by salinity variations and to validate the experimental procedures. For example, we could use the sodium-potassium ATPase (Na^+^/K^+^-ATPase *α*-subunit), a membrane protein which maintains ion gradients required for cell homeostasis, to assess the osmoregulatory status of fish in natural environments. In this study, we did not use a positive control gene because we have previously demonstrated that the mRNA expression levels of the Na^+^/K^+^-ATPase *α*-subunit in the same populations were correlated with the environmental salinity [43].

### 2.6. RNA Extraction, mRNA Purification, Reverse Transcription, and Real-Time PCR Analysis

Total RNA was extracted with a TRIZOL® reagent (Gibco-BRL, USA) according to the manufacturer's instructions. RNA concentrations were determined spectrophotometrically, and RNA integrity was verified by 1% TAE 1X agarose gel electrophoresis (40 mM Tris, acetate, and 1 mM EDTA). The relative expression of SLC4A1 (GenBank accession number ES881098), MAPK (ES881524), and LDH (ES881483) was analysed in both natural and experimental populations.

The eukaryotic mRNAs possess a repeat of adenosine residues at their 3′ end, which is used in the purification method of the Poly(A)Purist™ kit (Ambion). This method consists firstly in passing a total RNA solution onto a column having binding sites which are polydT oligonucleotides. These oligo (dT) are trapped in magnetic particles coupled to streptavidin and retain only the mRNAs. The aqueous phase which contains the ribosomal RNAs and the transfer RNAs is eliminated, and then, the mRNAs are eluted with ultrapure water previously autoclaved. We used this method to purify the mRNA from 2 mg of total RNA for both experimental and wild population samples.

The purified mRNAs of the gill from both experimental and wild populations are retrotranscribed into cDNA using the BD PCR-Select™ cDNA Subtraction Kit (Ambion). To synthesize the first strand of cDNA, 4 *μ*g of mRNA was mixed with 1 *μ*L of cDNA Synthesis Primer in a total volume of 5 *μ*L. The whole mixture is heated at 70°C for 2 minutes to eliminate potential secondary structures. After denaturation, the mixture is immediately stabilized by cooling in ice. Then, 2 *μ*L of 5X Fist-Strand Buffer, 1 *μ*L of dNTP Mix (10 mM each), 1 *μ*L of sterile water, and 1 *μ*L of AMV Reverse Transcriptase (20 *μ*L/*μ*L) are added to this reaction mixture. This final mixture is incubated at 42°C for 1 h and 30 min in a PTC-100MT thermocycler (MJ Research Inc.). The second strand of cDNA is synthesized by adding the following products to the tubes containing the first-strand synthesis reaction: 48.4 *μ*L of sterile water, 16 *μ*L of 5X Second-Strand Buffer, 1.6 *μ*L of dNTP Mix (10 mM), and 4 *μ*L of 20X Second-Strand Enzyme Cocktail. The total reaction is incubated at 16°C for 2 h and 30 min, and 2 *μ*L of the T4 is added 30 min before the end of the incubation. The synthesis of the second strand is stopped by adding 4 *μ*L of 20X EDTA/Glycogen Mix. The double-stranded cDNA is purified by phenol/chloroform extraction, precipitated with 100% ethanol, washed with 75% ethanol, and suspended into 50 *μ*L of nuclease-free water.

Primer3 software was used to design primers to amplify these genes.

The following forward (F) and reverse (R) primer sequences were used for qRT-PCR amplification: SLC4A1 (F: TCTGCAAAGAAGTGGCATCA; R: ATGACGCCAAGGTGACATTT), LDH (F: TGATCACCTCGTAGGCACTG; R: AAATGTGGCTGGAGTCAACC), MAPK (F: CTGGCCCTTCAACAGAGACTG; R: CTCTTCGATGGCCTGTTTCAC), and beta-actin (F: ACAGGTCCTTACGGATGTCG; R: CTCTTCCAGCCTTCCTTCCT).

Gene expression was quantified using quantitative real-time PCR (qRT-PCR) on a LightCycler (Roche Molecular Biomedicals) using the QuantiTect SYBR Green PCR Master Mix kit (Qiagen). Quantification of each sample was performed in a total volume of 10 *μ*L containing 1 *μ*L of cDNA, 0.5 *μ*L of each primer, and 1X of SYBR Green Master Mix (Qiagen). Each qRT-PCR reaction was conducted in duplicate, with an initial denaturation step of 15 min at 95°C, followed by an amplification of the target cDNA for 40 cycles, each cycle consisting of denaturation at 95°C for 15 s, annealing between 54°C and 55°C for 15 s, and elongation at 72°C for 15 s. To determine qRT-PCR efficiency of each primer pair, standard curves were generated using five serial dilutions (1, 1/10, 1/50, 1/ 100, and 1/500) of a unique cDNA sample constituted of a pool of 6 cDNA from each salinity group or population to be analysed. The efficiencies (*E*) of qRT-PCR reactions were calculated from the given slope of the standard curve according to the equation *E* = 10^(−1/slope)^. The amplification products were validated by analysing the amplicon size by means of agarose gel electrophoresis. Results are shown as changes in relative expression, normalised to the reference gene, *β*-actin, using the 2^−ΔΔCt^ method described by Kultz [34]; *β*-actin was previously analysed and showed no change in response to salinity acclimation.

### 2.7. Statistical Analyses

Gene expression data at each site were expressed as mean ± SEM. Bartlett [44] and Kolmogorov-Smirnov [45] tests were, respectively, used to test for homogeneity of variances and normality of the data. These tests showed that expression data were not normally distributed and did not have uniform variance. Therefore, only nonparametric statistical tests were applied. For each variable, a Kruskal-Wallis nonparametric analysis of variance (ANOVA) [46] was performed to reveal significant differences in means between salinity conditions and the sampling sites. The Mann–Whitney *U* test was applied as a post hoc test to assess differences in gene expression levels between salinity conditions and populations. Using the individual data from the sites, the correlation between salinity, temperature, and mRNA transcription levels of all genes was assessed by means of Spearman's rank correlation test [43]. These tests were performed with R Software (v.3.1.1). For all tests, a significance level of 0.05 was applied.

## 3. Results

### 3.1. Environmental Data

The salinity measured at the sampling sites during the dry season ranged from 0 to 100 psu (Table 2). Salinities at the reference sites ranged from 0 (Guiers Lake, freshwater) to 37 (Hann Bay, seawater). The salinity was markedly higher at all the sites of the Saloum estuary, up to an extreme of 100 psu at Kaolack (Table 2). The peculiarities of the study area are seasonal changes in ambient salinity and associated deterioration of water quality due to the alternation of an extended eight-month dry season (November to June) and a short four-month rainy season (July to October). In the Gambia and Saloum estuaries, salinity levels change very significantly between these two seasons [28, 29]. The salinity increased very significantly during the first months following the end of the rainy season because of intense evaporation [6]. It becomes then quite stable at the end of the dry season. During the short rainy season, by contrast, salinity in the estuaries is very unstable and can decrease considerably due to inputs of freshwater from precipitation, which drastically changes the water quality (turbidity). The poorer water quality during the rainy season represents an additional abiotic factor that could be constraining for the populations of *S. melanotheron*. However, the fish analysed in this study were collected at the end of the dry season (June) when the salinity in the estuaries had been stable for some months and the water turbidity was at the lowest level. The water temperature (Table 2) varied slightly among the sites, ranging from 26 to 29°C. The additional environmental data indicated that salinities measured at all the sites of the Saloum estuary for combined seasonal data ranged from brackish to hypersaline (up to 130 psu) in the estuary's upper reaches (Supplementary Figure 1). At Guiers Lake, freshwater conditions prevailed throughout the year whereas at Hann Bay, salinity varied slightly between 37 and 38 psu (Supplementary Table 1). Water temperatures in the Saloum and Gambia estuaries showed slight variations (Supplementary Figure 2). Average water temperatures were 24.9 and 25°C at Guiers Lake and Hann Bay, respectively (Supplementary Table 1). None of the sampling locations did reach values of percentage oxygen saturation that are limiting to the survival of fish (Supplementary Figure 3 and Table 1). Based on all these results, it can be concluded that salinity is the most constraining environmental factor for *S. melanotheron* in our study area during the dry season.

### 3.2. Branchial Abundance of SLC4A1, MAPK, and LDH mRNA

**Figure 2:**
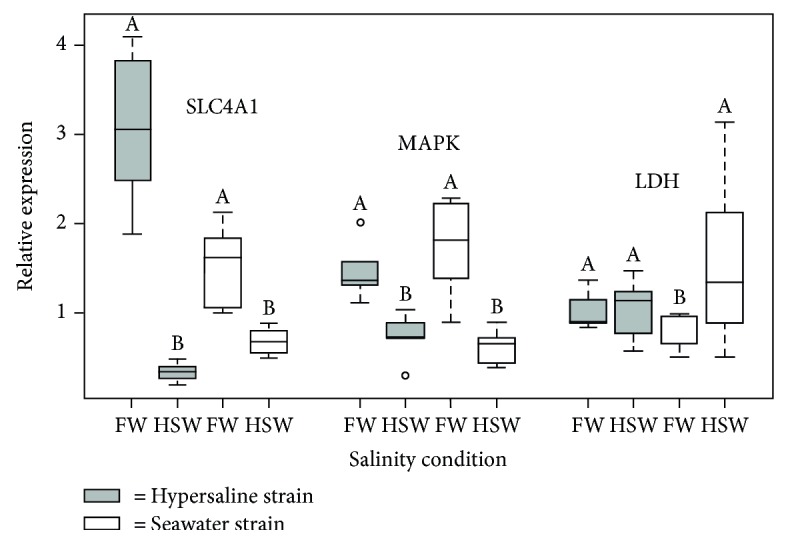
Relative expression of SLC4A1, MAPK, and LDH in fish gills. The relative expression was measured after an exposure period of 45 (hypersaline strain) or 10 (seawater strain) days to freshwater (FW) and hypersaline water (HSW). Data are illustrated as boxplots with the median represented by a horizontal line and the 25th and 75th percentiles corresponding to the bottom and top edges of the boxes. Different letters on the boxplots indicate significant difference in gene expression levels whereas the same letter indicated that there is no significant difference.

The efficiency of the real-time PCR analysis showed values around 1.9, which implies that the two primer pairs of each gene (including the reference gene, *β*-actin) amplified a single specific product. A melting curve analysis and agarose gel visualization are not shown in this study, but the products of amplification of the reference gene were previously validated by analysing the amplicon size on agarose gel electrophoresis [47]. Relative abundance in individuals from both hypersaline water and seawater strains was calculated to correct for differences in efficiency. The results revealed significant differences in SLC4A1 and MAPK expression levels between salinity conditions (Kruskal-Wallis test; *p* < 0.05) in the hypersaline water strain. Fish maintained in freshwater had significantly higher SLC4A1 mRNA levels than those acclimated to hypersaline water (Figure 2). The MAPK mRNA levels exhibited a similar pattern between salinity conditions, being higher in freshwater and lower in hypersaline water (Figure 2). The seawater strain (Joal) showed significant differences in LDH expression levels between freshwater and hypersaline water (Kruskal-Wallis test; *p* < 0.05), but there was no significant difference between these two salinity conditions in individuals of the hypersaline (Saloum) strain (Figure 2). The comparisons between strains showed that the SLC4A1 and MAPK expression profiles are comparable between the strains (Figure 2) with elevated levels in freshwater and low levels in hypersaline water. By contrast, there are significant differences in gene expression profiles between the strains for the LDH gene with elevated levels being recorded in hypersaline water for the seawater strain.

**Figure 3:**
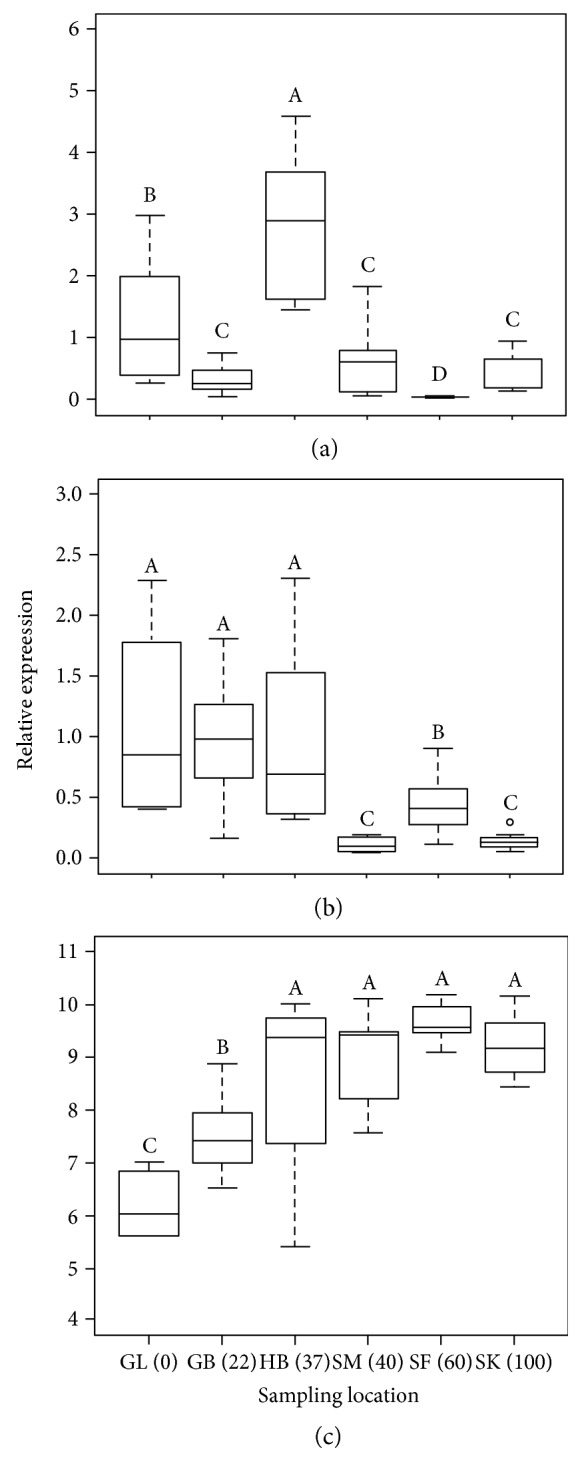
Relative expression of SLC4A1 (a), MAPK (b), and LDH (c) in six wild populations adapted to different salinities. Data are illustrated as boxplots with the median represented by the horizontal line and the 25th and 75th percentiles corresponding to the bottom and top edges of the boxes. Different alphabetic letters above the boxplots indicate significant difference in gene expression levels whereas the same letter indicated that there is no significant difference. GL: Guiers Lake; GB: Gambia Balingho; HB: Hann Bay; SM: Saloum Missirah; SF: Saloum Foundiougne; SK: Saloum Kaolack. The values in brackets on the *x*-axis correspond to salinities recorded at each sampling location at the time of fish sampling.

In wild populations, the comparison of SLC4A1 expression between sampling locations revealed significant differences between populations (Kruskal-Wallis test; *p* < 0.05). The SLC4A1 mRNA levels were higher at site 3 (Figure 3(a)) than at the sampling sites of the Gambia (site 2) and Saloum (4a, 4b, and 4c) estuaries, and they were also higher than those at site 1, while the levels at this latter site were higher than those at the other sampling locations. Within the Saloum estuary, fish sampled at site 4b, whose salinity was intermediate, had lower SLC4A1 mRNA levels than fish sampled at the less saline station (4a) and the most saline sampling site (4c) (Figure 3(a)). No significant difference in SLC4A1 expression was found between sites 4a and 4c. The amounts of MAPK mRNA were significantly higher (Kruskal-Wallis test; *p* < 0.05) in freshwater, brackish water, and seawater at sites 1, 2, and 3, respectively (Figure 3(b)), in comparison to the most saline sampling sites (4a, 4b, and 4c) of the Saloum estuary. No significant difference in MAPK expression was found between sites 1, 2, and 3 (Figure 3(b)). Within the Saloum estuary, the MAPK expression levels were significantly higher at site 2 in comparison to sites 1 and 3, but there were no significant differences between these two locations. The highest LDH mRNA levels (Kruskal-Wallis test; *p* < 0.05) were recorded at site 3 and the Saloum estuary (4a, 4b, and 4c) whereas the lowest levels were observed at the least saline locations, sites 1 and 2 (Figure 3(c)). Within the Saloum estuary, there was no significant difference in LDH expression between locations. The relative expression levels of LDH at the Saloum estuary locations were not significantly different from those at site 3 (Figure 3(c)). The amounts of LDH mRNA at site 2 were higher than those at site 1.

### 3.3. Correlation between Salinity, Temperature, and Gene Expression

**Figure 4:**
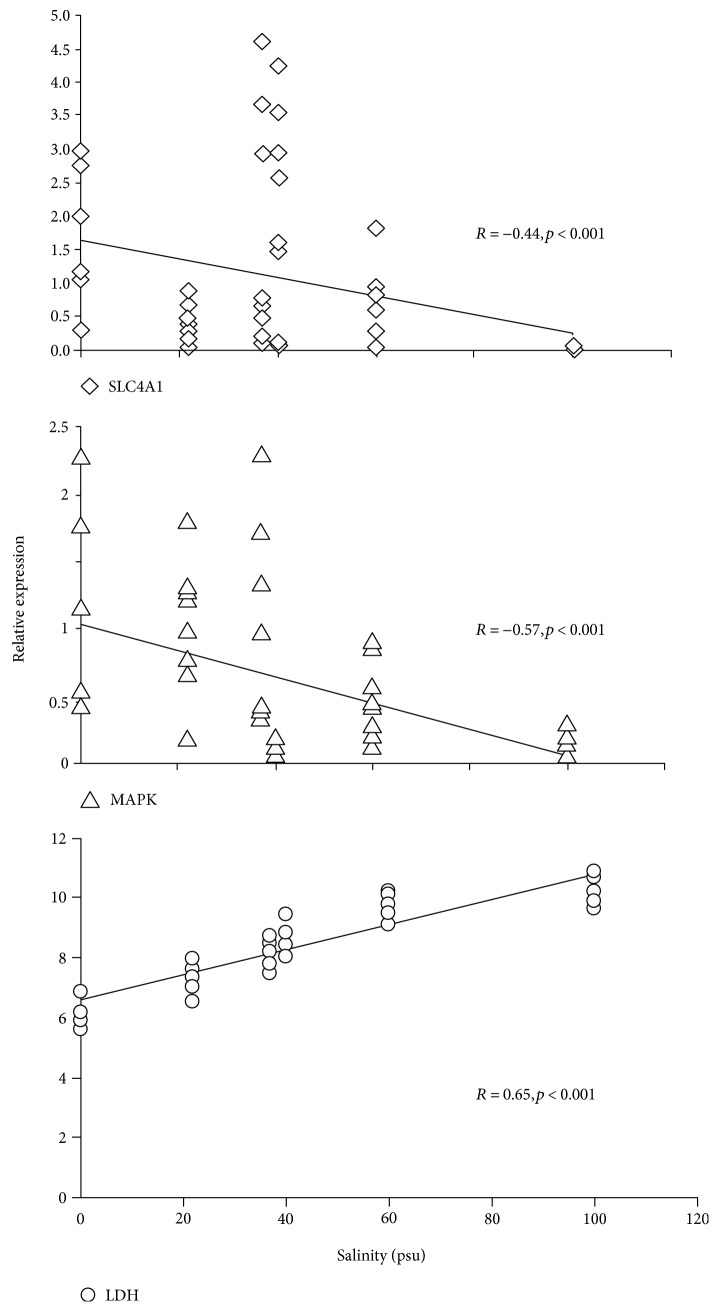
Relationship between environmental salinity and mRNA levels of SLC4A1, MAPK, and LDH. The mRNA expression levels represent the relative expression normalised to *β*-actin.

Environmental salinity and SLC4A1 mRNA levels were negatively correlated (Spearman's rank correlation: *R* = −0.44, *p* < 0.001), and the same relationship was found for salinity and MAPK relative expression (*R* = −0.57, *p* < 0.001) (Figure 4). In contrast, LDH mRNA levels were significantly positively correlated with salinity (*R* = 0.65; *p* < 0.001) (Figure 4). There was no significant correlation between ambient temperature and mRNA levels of SLC4A1 (*R* = 0.033; *p* > 0.5), MAPK (*R* = 0.029; *p* > 0.5), and LDH (*R* = −0.31; *p* > 0.5).

## 4. Discussion

The results of the experiments conducted under controlled laboratory conditions revealed significant differences depending on the salinity conditions at which fish were held. In contrast, the differential expression in the natural environment is not consistent with that in the model of neutral divergence of wild populations and also does not reflect salinity-induced differences in life history traits observed between populations. These results suggest that the gene expression patterns are not a result of evolutionary forces such as genetic drift or adaptation by natural selection. They suggest that genes responded to various environmental stressors, which may occur in combination with biotic stressors, including factors intrinsic to animals. Although gene expression variations were explored in two different strains (seawater and hypersaline water strains) established in the laboratory 15 years ago under experimental conditions identical to those described above, overall genes showed similar expression patterns. These results indicate a reproducibility but also an absence of a strain effect. By contrast, the lack of correlations between laboratory and wild conditions may reflect the adaptation of experimental fish to laboratory conditions. It is likely that individuals adapted in the laboratory for such a long time have evolved particular adaptations to the laboratory conditions plus the effect of inbreeding.

For a better understanding of how the differential expression observed here could relate to the adaptation of tilapia populations to environmental salinities, it is necessary to contrast gene expression patterns with the genetic structure of the populations inferred from neutral markers. The expectation for such comparisons is whether gene expression patterns are correlated with this genetic differentiation; this would probably indicate that differences among populations have arisen from genetic drift. The genetic divergence among populations was not analysed in this study, but previous work has shown that populations of *S. melanotheron* are strongly structured, even at microgeographical scales (i.e., within the Saloum estuary) [48]. This high-population genetic structure is essentially due to the low larval dispersal ability of this species, which results from its mouth-brooding reproductive behaviour. However, the patterns of gene expression found here do not reflect the genetic differentiation of *S. melanotheron* populations revealed by neutral markers. For example, the genetically and geographically remote populations of Saloum sites 4a, 4b, and 4c did not display any difference in SLC4A1 expression levels. Likewise, no significant difference in LDH expression levels was found between populations of sites 1 and 2, two populations that are genetically strongly differentiated. These observations indicate that the differential expression observed in this study is not the result of genetic drift. By contrast, it cannot be ruled out that this differential expression reflects the effects of natural selection, which could sort out some specific adaptive polymorphisms that may increase the relative fitness of populations in their natural environments. However, considering the extensive temporal variations of salinity conditions in the Saloum and Gambia estuaries throughout the year, the idea that divergent selection could lead to fixation of mutations seems unlikely. Indeed, the salinity in the Saloum and Gambia estuaries increases during the first months following the end of the rainy season, because of intense evaporation [6], and then becomes quite stable at the end of the dry season. Thus, during the long dry season, the estuary only exhibits minor variations in salinity. By contrast, during the short rainy season, salinity in the estuary is very unstable and can decrease considerably due to inputs of freshwater from precipitation. In such a situation, long-term adaptation as the response to environmental conditions that remain relatively constant throughout the lifetime of the species is unlikely. By contrast, unstable salinity in the rainy season may represent a major stress factor that might drive selection of genotypes better suited to adjusting physiological responses to manage the consequences of stress.

Thus, the differences in gene expression between populations may result from gene plasticity in response to salinity variations. The expression of genes analysed here may be differentially regulated to respond directly to the salinity variation [49]. Therefore, it would be interesting to analyse and compare the epigenetic patterns and diversity between populations to explain differential responses in terms of gene expression to the environment. Environmentally induced phenotypic variations, known as gene expression plasticity, have been reported in several fish species [50–52]. This plasticity can be adaptive when the phenotypic variation confers higher fitness to a particular individual [53, 54]. Previous studies have shown that *S. melanotheron* populations exhibit greater fitness at intermediate salinities, where the energy cost for osmoregulation is lower compared to that at hypersaline conditions, where impaired growth and precocious reproduction were observed [28, 55]. However, differential expression patterns observed here do not reflect these differences in life history traits induced by salinity variations. Moreover, the correlations between gene expression levels and environmental salinity are somewhat ambiguous, suggesting the potential influence of factors other than ambient salinity.

The SLC4A1 gene may be induced by other factors including anthropogenic pollution, because this gene is involved in other regulatory processes, such as metal homeostasis. Likewise, the LDH expression may reflect switches in metabolic pathways used to yield the energy required for hydromineral maintenance [56–58]. Indeed, elevated LDH expression levels were seen in hypersaline water for the seawater strain, which is consistent with the elevated levels observed in all the wild populations where salinities are above 22 psu. These results point to the higher energy requirements. While the elevated LDH expression levels in the Saloum estuary (sites 4a, 4b, and 4c) may reflect energy cost for osmoregulation at high salinities, the elevated mRNA amounts observed in seawater (site 3) are somewhat difficult to explain. They may reflect energy investments in growth since *S. melanotheron* is known for having the best growth performances in seawater [55]. Similarly, elevated expression levels in seawater (site 3) for the SLC4A1 are somewhat difficult to explain. They may reflect an involvement of this gene in somatic growth, especially since the species has better growth rates in salinities around seawater. Indeed, it has been demonstrated that the MAPK/ERK pathway activation is inhibited during fasting and stimulated during refeeding [59]. The same authors have established a link between insulin-like growth factor-I (IGF-I), the activation of the MAPK/ERK, and somatic growth in fine flounder, strongly suggesting regulatory effects of MAPK pathways on fish growth. While the low MAPK expression levels when salinities are above normal seawater salinity, only observed in the Saloum populations where impaired growth was observed [28, 55], are consistent with this interpretation, the low expression in brackish water (site 2, 22 psu) indicates quite the opposite. MAPK pathways are activated by changes in biotic factors including oxidative stress, growth factors, and cytokines [60, 61]. These internal cues are known to play important roles in transcriptional responses through feedback mechanisms, through which organisms respond to internal physiological state including nutrient and energy availability, growth rate, and pathogen infection, by activating gene expression programs. Mechanisms that directly link gene expression with internal variables differ from responses to external signals, through which gene expression is modulated directly by signal transduction pathways that translate extracellular stimuli into intracellular responses to cope with environmental variations. In natural environments where biotic factors cannot be controlled, their effects may interfere with external environmental factors and impact gene expression levels between populations. This may explain the absence of significant correlations between gene expression and ambient salinity in this study. It has been reported that many genes regulated during a stress response are not specifically induced by a given perturbation, but from a part of stress response pathways. Such unspecific stress response genes respond to various environmental stressors that may occur together and/or in combination with factors intrinsic to animals whose effects are more difficult to evaluate. Furthermore, unspecific stress response genes are also generally involved in a wide diversity of other biological functions including growth, sexual maturation, and reproduction, which may interfere with stress responses. The fact that the effects of biotic factors may interfere with external environmental factors in natural environments may explain the differences in LDH expression levels between the breeding strains and wild fish.

The main drawback of our approach is the limited number of genes analysed, but these genes are used here as proxies of the entire networks of stress response pathways. The roles of the SLC4A1 and MAPK genes have previously been investigated in the same populations in the context of adaptive plasticity of gene expression in response to salinity changes [17]. Reanalysing these genes here to address a new question has the advantage of better elucidating their adaptive roles in response to salinity variations. The experimental populations analysed here were exposed to specific treatments for different lengths of time, which may render the findings difficult to interpret with respect to population and salinity of origin and time of exposure to a particular treatment. However, both experiments indicated that two of the three genes analysed were significantly differentially expressed between the freshwater and hypersaline treatments, and in both cases, the population origin and therefore the salinities at which the fish were captured did not significantly affect the expression of the three genes. Likewise, differences between experiments in developmental stages, type of the tanks, food type, light, and time of the year do not seem to affect the fishes' physiology and, consequently, their levels of gene expression.

## 5. Conclusion

Genes that contribute to the specific traits for stress tolerance have been successfully studied and are still being used as biomarkers for investigating the adaptive mechanisms employed by living organisms to environmental variations. The relationship between gene expression and variation in environmental salinity in *S. melanotheron* populations along salinity gradients has been successfully explored in previous studies [17, 47, 62]. Significant correlations were found in populations between salinity and mRNA transcription levels of genes putatively involved in osmoregulation, suggesting that they play a role in salinity acclimatisation. It was also demonstrated that gene expression patterns in wild populations match salinity-related differences in life history traits previously observed in the same populations [63]. These results clearly indicated that *S. melanotheron* is an excellent model species for understanding the molecular and physiological mechanisms used by fish to cope with extreme changes in environmental salinities, particularly in the Senegal and Gambia estuaries where other factors, such as water temperature and dissolved oxygen, show considerable variations. These results also clearly demonstrate that candidate gene approaches can be applied to wild populations to identify genes and pathways involved in the acclimatisation to extreme salinities, including populations inhabiting estuarine ecosystems where the complexity of environmental conditions can pose great difficulties in the interpretation of gene expression profiles. The results of the present study show that candidate genes selected to be responsive to salinity variation under experimental conditions do not show the expected behaviour when monitored in wild samples naturally experimenting life in a similar salinity gradient. These results raise the possibility that the investigated genes are not specifically and exclusively responsive to salt stress. They can be qualified as unspecific stress response genes that responded to various environmental stressors which may occur in combination with biotic stressors including factors intrinsic to animals and whose effects are difficult to evaluate in wild populations. This study is among the first to illustrate the complexity of gene expression analyses in wild fish populations due to the inherent limitation of gene candidate identification approaches, which essentially rely on the knowledge in physiological, biochemical, and metabolic functions. These results are particularly significant because they provide an indication on how much attention needs to be paid when selecting candidate genes for studies on natural populations.

## Supplementary Material

Supplementary Figure 1: Evolution of surface and bottom water salinity in the Saloum and Gambian estuaries.
Supplementary Figure 2: Evolution of surface water temperature in the Saloum and Gambian estuaries.
Supplementary Figure 3: Evolution of surface and bottom % dissolved oxygen of surface and bottom waters in the Saloum and Gambian estuaries.
Supplementary Table 1: Additional environmental data from Guiers Lake and Hann Bay.

## Figures and Tables

**Table 1:** Sample characteristics of the black-chinned tilapia *Sarotherodon melanotheron* strains kept under different experimental conditions.

Strain	Strain origin	Experimental condition	Salinity (psu)	WT (°C)	NI
Kaolack	Upstream Saloum estuary (48 psu)	Freshwater	0	25	6
		Hypersaline water	70	25	6
Joal	Atlantic Ocean (38 psu)	Freshwater	0	25	6
		Hypersaline water	70	25	6

NI: number of individuals; WT: water temperature.

**Table 2:** Sample characteristics of the black-chinned tilapia *Sarotherodon melanotheron* from six wild populations acclimatised to different salinities that prevailed in their habitats.

Ecosystem	Station	Site number	GPS coordinates	Salinity (psu)	WT(°C)	NI	FL range (mm)	W range (g)
Guiers Lake	Keur Momar Sarr	1	16°15′00″N, 15°50′00″W	0	28	10	120–149	32–45
Gambia	Balingho	2	13°29′00″N, 15°37′00″W	22	29	10	122–152	42–60
Hann plage	Hann Bay	3	14°43′16″N, 17°26′13″W	37	28	10	124–161	41–80
Saloum estuary	Missirah	4a	13°40′60″N, 16°30′01″W	40	28	10	135–165	40–92
Saloum estuary	Foundiougne	4b	14°08′00″N, 16°28′00″W	60	28	10	122–145	40–52
Saloum estuary	Kaolack	4c	14°11′00″N, 16°15′00″W	100	26	10	123–147	33–51

WT: water temperature; NI: number of individuals; FL: fork length; W: weight.

## Abbreviations

SLC4A1: Band 3 anion transport protein
MAPK: Mitogen-activated protein kinase
LDH: Lactate dehydrogenase
qRT-PCR: Quantitative real-time PCR
SEM: Standard error of mean
SSH: Suppressive subtractive hybridisation.

## Additional Points

*Availability of Supporting Data*. All datasets supporting the results of this article are within the article and its Additional File 1. The accession numbers of any nucleic acid sequences of the three candidate markers used in this study are indicated in Section 2.6: SLC4A1 (GenBank accession number ES881098), MAPK (GenBank accession number ES881524), and LDH (GenBank accession number ES881483). *Authors' Information*. Mbaye Tine is an evolutionary biologist who performed his Doctorate Thesis in Evolutionary Biology and Ecology at the University of Montpellier II, in the department Integrative Biology of the Institute of Sciences and Evolution, under the direction of Dr. François Bonhomme (CNRS) and Dr. Jean-Dominique Durand (IRD). His research focused on genetic and physiological mechanisms underlying the adaptation of fishes to environmental variations including changes in ambient salinity. He performed his first postdoctoral research in Genomics and Evolution in the laboratory of Dr. Richard Reinhardt at the Max Plank Institute for Molecular Genetics in Berlin. Afterward, he performed a second postdoc in Genomics and Next Generation Sequencing at the Genome Centre at MIP Cologne. More specifically, he worked on the sequencing project of the European sea bass (*Dicentrarchus labrax* L.) genome, which was published in Nature Communication. He is currently doing his research at the Molecular Zoology Laboratory at Johannesburg University where he works on a project that aims at investigating thermal adaptation and the role of phenotypic plasticity in coastal invertebrates and/or fishes by studying populations of species whose ranges span across multiple South African marine biogeographic provinces.
