# Supplementary material for: Evidence of the Complexity of Gene Expression Analysis in Fish Wild Populations
Source: Int J Genomics. 2017 Oct 23;2017:1258396. doi: 10.1155/2017/1258396 (PMC5672613; doi:10.1155/2017/1258396)
Supplement: Supplementary file 1 — Supplementary Figure 1: Evolution of surface and bottom water salinity in the Saloum and Gambian estuaries. Supplementary Figure 2: Evolution of surface water temperature in the Saloum and Gambian estuaries. Supplementary Figure 3: Evolution of surface and bottom % dissolved oxygen of surface and bottom waters in the Saloum and Gambian estuaries. Supplementary Table 1: Additional environmental data from Guiers Lake and Hann Bay. [file 1258396.f1.pdf]

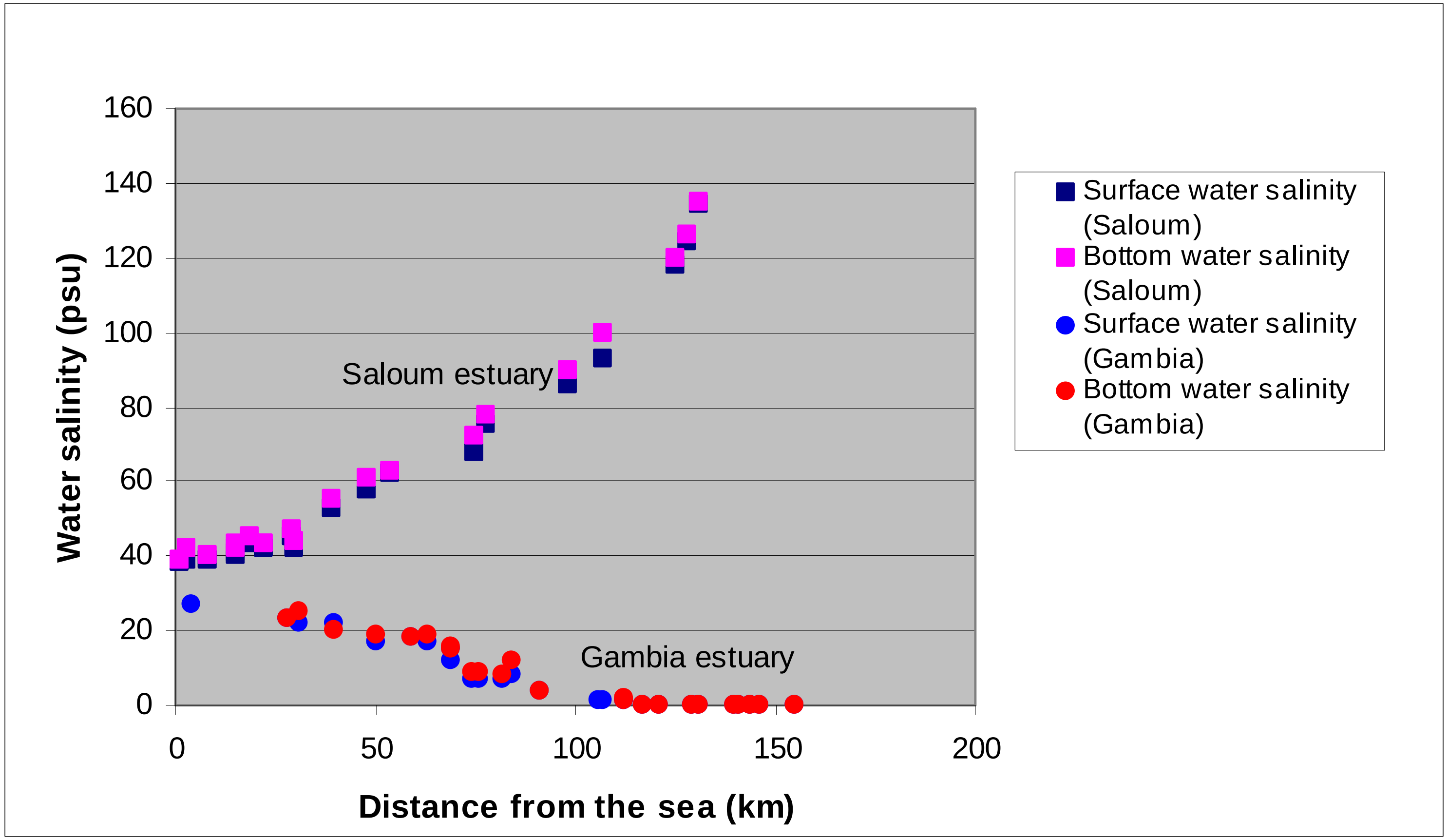

Supplementary Figure 1: Evolution of surface and bottom water salinity in the Saloum and Gambian estuaries

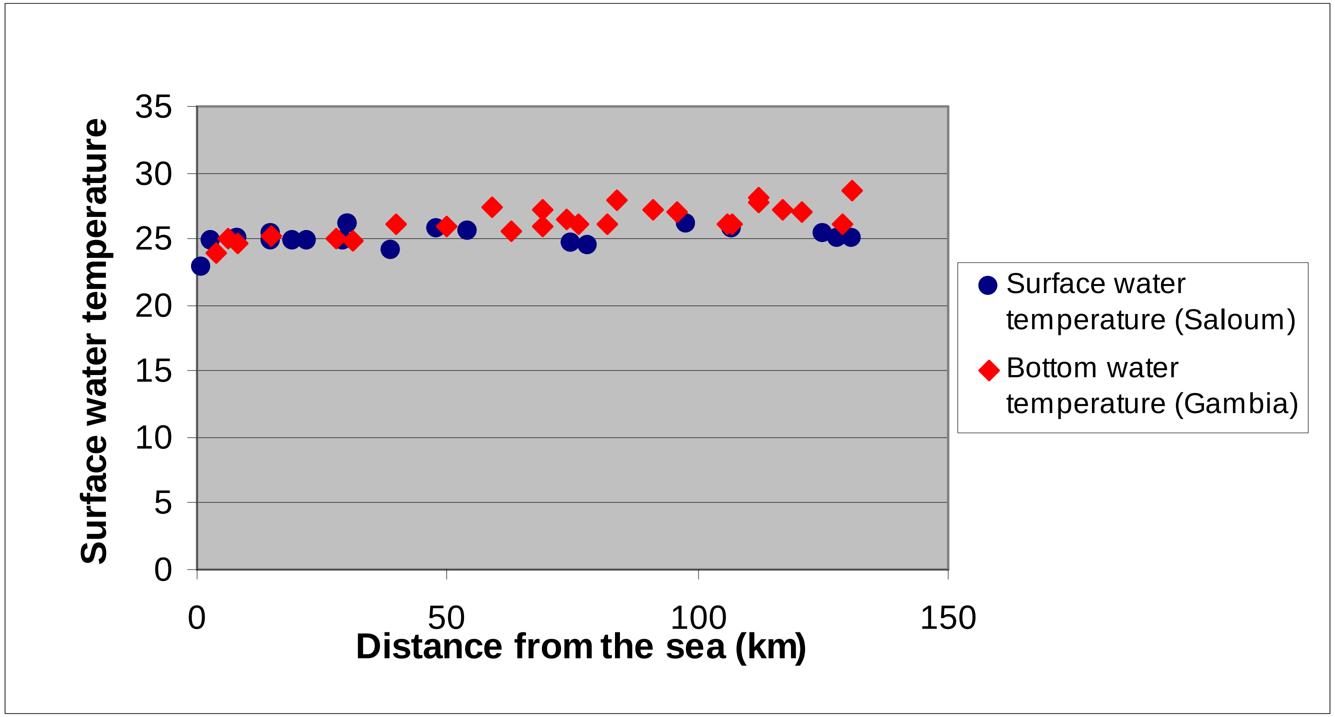

Supplementary Figure 2: Evolution of surface water temperature in the Saloum and Gambian estuaries.

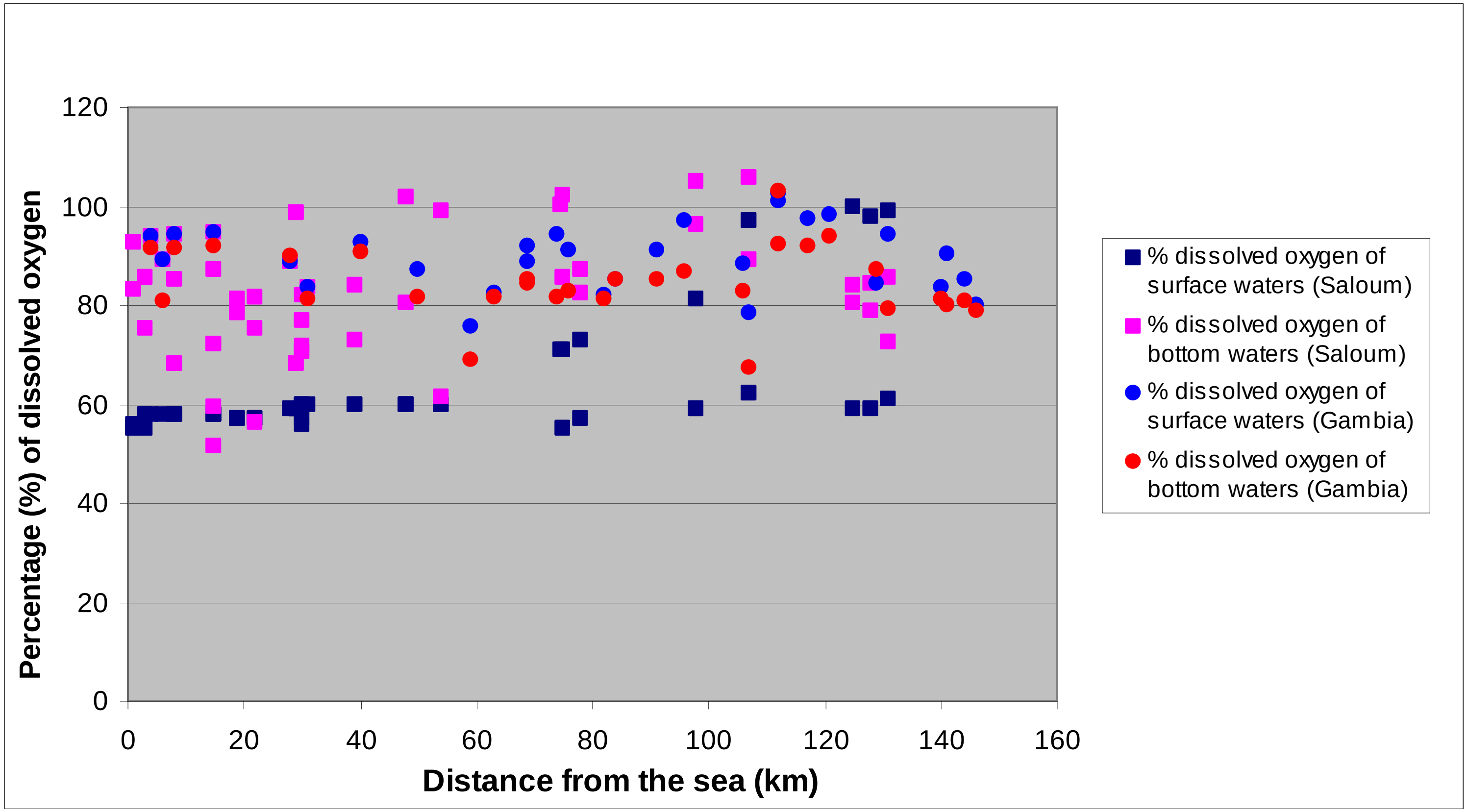

Supplementary Figure 3: Evolution of surface and bottom % dissolved oxygen of surface and bottom waters in the Saloum and Gambian estuaries.

Supplementary Table 1: Additional environmental data from Guiers Lake and Hann Bay

Variable	**Guiers Lake**	**Hann Bay**
Salinity (psu)	0	37-38
Water temperature (℃)	24.9	25
Oxygen (mg L^{-1})	6.7	4.84
